# Research into Prediction Method for Pressure Pulsations in a Centrifugal Pump Based on Variational Mode Decomposition–Particle Swarm Optimization and Hybrid Deep Learning Models

**DOI:** 10.3390/s24134196

**Published:** 2024-06-27

**Authors:** Jiaxing Lu, Yuzhuo Zhou, Yanlong Ge, Jiahong Liu, Chuan Zhang

**Affiliations:** 1 Key Laboratory of Fluid and Power Machinery, Ministry of Education, Xihua University, Chengdu 610039, China; 2 Key Laboratory of Fluid Machinery and Engineering, Xihua University, Chengdu 610039, China; 3 School of Energy and Power Engineering, Xihua University, Chengdu 610039, China; 4 PowerChina Hydropower Development Group Co., Ltd., PowerChina, 139 Tianfu Second Street, Chengdu 610041, China

**Keywords:** centrifugal pump, pressure pulsation, deep learning, modal decomposition, prediction

## Abstract

Centrifugal pump pressure pulsation contains various signals in different frequency domains, which interact and superimpose on each other, resulting in characteristics such as intermittency, non-stationarity, and complexity. Computational Fluid Dynamics (CFD) and traditional time series models are unable to handle nonlinear and non-smooth problems, resulting in low accuracy in the prediction of pressure fluctuations. Therefore, this study proposes a new method for predicting pressure fluctuations. The pressure pulsation signals at the inlet of the centrifugal pump are processed using Variational Mode Decomposition–Particle Swarm Optimization (VMD-PSO), and the signal is predicted by Convolutional Neural Networks–Long Short-Term Memory (CNN-LSTM) model. The results indicate that the proposed prediction model combining VMD-PSO with four neural networks outperforms the single neural network prediction model in terms of prediction accuracy. Relatively high accuracy is achieved by the VMD-PSO-CNN-LSTM model for multiple forward prediction steps, particularly for a forward prediction step of 1 (Pre = 1), with a root mean square error of 0.03145 and an average absolute percentage error of 1.007%. This study provides a scientific basis for the intelligent operation of centrifugal pumps.

## 1. Introduction

The pressure pulsation characteristics of unstable flow within centrifugal pumps have been the subject of extensive research by scholars worldwide [1]. The highly intricate internal flow of centrifugal pumps gives rise to significant pressure pulsations [2]. In particular, the interaction between rotating and stationary components generates periodic hydraulic excitation forces, resulting in vibration and noise issues in centrifugal pumps [3]. The pressure pulsations in the internal flow field of centrifugal pumps serve as the primary cause of operational instability [4]. They not only induce resonance in the pipeline system but also impose cyclic loading on precision components, leading to fatigue failure of various pump components [5,6]. Therefore, gaining a precise understanding of the distribution characteristics of pressure pulsation is crucial for enhancing the vibration suppression and operational stability of centrifugal pumps [7]. Furthermore, accurate prediction of pressure pulsation under normal operating conditions and the implementation of corresponding mitigation measures hold significant importance in improving operational stability and extending the lifespan of centrifugal pumps [8,9].

Wang et al. [10] proposed a modified prediction model to quantitatively describe the relationship between bubble evolution inside Venturi tubes and excitation pressure pulsation. They validated the accuracy of the model through Large Eddy Simulation (LES) and improved the prediction accuracy of pressure pulsation. Jiang et al. [11] applied the k-means clustering algorithm to evaluate the health condition of hydraulic turbine units by finding the optimal value of *k*. They developed a pressure pulsation trend prediction model for hydraulic turbine units based on Long Short-Term Memory (LSTM) networks for time series analysis. Zhang [12] used the Advanced Modeling Environment for Simulation (AMESim R12) software to establish a functional model of gear pumps and obtain the pressure pulsation of gear pumps. They further developed a neural network model to predict pressure pulsation and verified the accuracy of the established neural network model by comparing the two prediction results. Zhu et al. [13] obtained the characteristics of vortex stripes in the draft tube of a Francis turbine and induced the pressure pulsation through steady and unsteady turbulent numerical simulations. Chen et al. [14] optimized the weights and thresholds of a Backpropagation Neural Network (BPNN) through the global searching ability of the Mind Evolutionary Algorithm (MEA). The optimized BPNN achieved a prediction accuracy of 99.148%, which was 0.721% higher than that of the traditional BPNN. Xu et al. [15] proposed a prediction conversion method for the inlet pressure pulsation of draft tubes based on the zero circulation unit flow and the blade outlet velocity head by comparing the experimental results of pressure pulsation in the draft tubes of 12 hydroelectric turbine models and similar prototype machines. Xu et al. [16] compared the prediction performance of Proper Orthogonal Decomposition (POD)–Long Short-Term Memory (LSTM) and Convolutional Autoencoder (CAE)–Long Short-Term Memory (LSTM) methods based on unsteady flow field data of a cylinder. They found that the prediction accuracy of POD-LSTM significantly decreased with an increase in time step, while CAE-LSTM maintained good robustness until the last time step. Lin et al. [17] used the Particle Swarm Optimization–Least Squares Support Vector Regression (PSO-LSSVR) surrogate model to optimize the design of two impellers in a hydrogenation feedstock multistage pump, targeting head and efficiency as optimization objectives. The results showed that the pump optimized with the PSO-LSSVR surrogate model reduced energy consumption by 9% at the rated flow. Li et al. [18] adopted a prediction model based on Particle Swarm Optimization–Support Vector Machine (PSO-SVM) to identify the cavitation state of shielded motor pumps. The experimental results demonstrated that the method effectively extracted cavitation features, reduced redundant components, and achieved high recognition accuracy and strong anti-interference capability. Rong et al. [19] combined the Design of Experiments method with a Radial Basis Function Neural Network (RBF). Through repeated design and simulation, the experimental results showed that, after optimization, channel vortices and hydraulic losses were effectively controlled, and the pump’s efficiency improved by 8.34%.

To sum up, none of the existing studies has proposed a systematic and reliable method for the prediction of pressure pulsation in centrifugal pumps. Given the complexity of the internal flow dynamics and the significant impact of pressure pulsations, traditional analytical methods often fall short of providing accurate predictions. Therefore, this study proposes a prediction model for centrifugal pump pressure pulsation based on PSO-VMD-CNN-LSTM. First, the parameters of VMD are optimized using the PSO algorithm. This optimization process allows for the adaptive decomposition of the pressure pulsation signals and eliminates uncertainties arising from manual parameter selection [20]. Next, each Intrinsic Mode Function (IMF) component obtained from the decomposition is fed into a hybrid neural network comprising CNN and LSTM networks. This configuration enables accurate prediction of each component signal [21]. Finally, the obtained results are aggregated to derive the final prediction of pressure pulsation. The findings demonstrate that the proposed PSO-VMD-CNN-LSTM model achieves higher accuracy when compared with other mathematical models. It facilitates the adaptive decomposition of pressure pulsation signals and effectively predicts the pressure pulsation at the inlet and outlet of centrifugal pumps.

## 2. Main Models and Algorithms

### 2.1. Variational Mode Decomposition

Variational Mode Decomposition (VMD) is a completely non-recursive signal processing method, and its decomposition process essentially solves a variational problem [22]. During the decomposition, the Intrinsic Mode Functions (IMFs) are defined as amplitude-modulated and frequency-modulated functions with bandwidth limitations. The specific steps are as follows:

Step 1: Initialize $\left\{v_{k}^{1}\right\}$, $\left\{\omega_{k}^{1}\right\}$, $\lambda^{1}$, and n=0.

Step 2: Enter the loop and set n=n+1.

Step 3: Update $\omega_{k}$ and $\omega_{k}$ according to the update formula until the number of decompositions reaches k, then stop the inner loop. The update formula is as follows:

(1) $$\omega_{k}^{n+1}=\frac{\int_{0}^{x}\omega \left|v_{k}^{n=1}\omega \right|d\omega }{\int_{0}^{x}{\left|v_{k}^{n=1}\omega \right|}^{2}d\omega }$$

(2) $$v_{k}^{n=1}=\frac{f(w)-\sum_{i\neq k}v_{i}(w)+\lambda (w)/2}{1+2\alpha {\left(\omega -\omega_{k}\right)}^{2}}$$

Step 4: λ is calculated according to the following formula:

(3) $$\lambda^{n+1}=\lambda^{n}(\omega )+y(f(w)-\sum_{k}v_{k}^{n+1}(\omega ))$$

If the accuracy meets the stopping condition:

(4) $$\sum_{k}{||v_{k}^{n+1}-v_{k}^{n}||}_{2}^{2}/{||v_{k}^{n}||}_{2}^{2}<\varepsilon$$

the loop is stopped. Otherwise, return to Step 2 and continue the loop.

In the above steps, λ represents Lagrange multipliers, k represents the number of components, n represents the iteration count, and α represents the penalty parameter. $\left\{\left.v_{k}\right\}\right.=\left\{\left.v^{1},v^{2}\ldots v^{k}\right\}\right.$ represents the decomposed IMF components, and $\left\{\left.\omega_{k}\right\}\right.=\left\{\left.\omega^{1},\omega^{2}\ldots \omega^{k}\right\}\right.$ represents the central frequencies of each component. γ represents the noise tolerance, and γ=0 can be set to achieve better denoising effects when the signal contains strong noise.

### 2.2. Particle Swarm Optimization

Particle Swarm Optimization (PSO) is a heuristic optimization technique based on the behavior of a particle swarm. The update is based on the particle’s previous best position (pbest) and the best position (gbest) among all particles. The specific update equations are as follows [23]:

(5) $$v_{i}(t+1)=wv_{i}+c_{1}r_{1}(pbest_{i}-p_{i}(t))+c_{2}r_{2}(gbest-p_{i}(t))$$

(6) $$p_{i}(t+1)=p_{i}(t)+v_{i}r(t+1)$$

where $r_{1}$ and $r_{2}$ are uniformly distributed random variables in the range [0, 1], $c_{1}$ and $c_{2}$ are positive acceleration coefficients associated with individual and social learning factors, and w is the inertia weight.

### 2.3. 1DCNN Prediction Model

**Figure 1:**
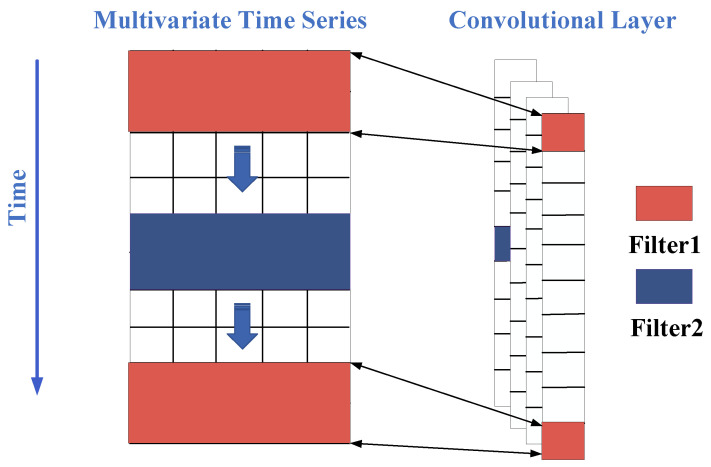
Schematic diagram of 1DCNN structure.

A Convolutional Neural Network (CNN) is a class of feedforward neural networks with a deep structure. In this study, 1DCNN was used to process the time series data of pressure pulsation [24]. The analysis process of 1DCNN is illustrated in Figure 1, which shows a multidimensional matrix of the input time series data. The red and blue colors represent different filters [25]. After the filters perform convolutional operations with the input data, the extracted feature dimension becomes N × 1, where N depends on the input data dimension, filter size, and convolution stride. Assuming M filters are used, the final extracted feature dimension becomes N × M [26].

### 2.4. TCN Prediction Model

**Figure 2:**
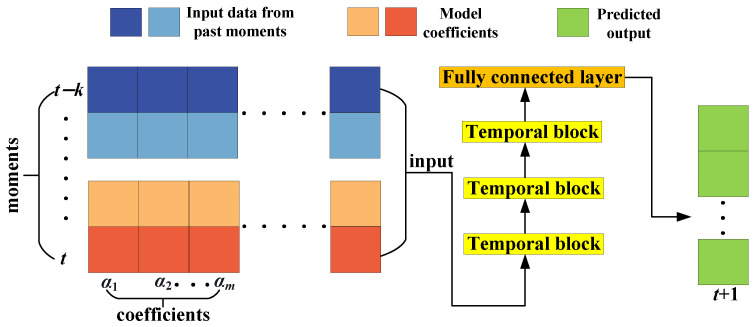
Schematic diagram of TCN structure.

A Temporal Convolutional Network (TCN) is a deep learning model used for sequence modeling, and its basic structure is illustrated in Figure 2. TCN’s convolutional layers not only handle input and feature extraction but also employ techniques such as causal convolution and dilated convolution. Causal convolution ensures that the model does not utilize future information during prediction and only relies on past and current inputs. Dilated convolution, on the other hand, increases the receptive field of the convolutional kernel, allowing it to capture longer-term dependencies [27].

### 2.5. LSTM Prediction Model

**Figure 3:**
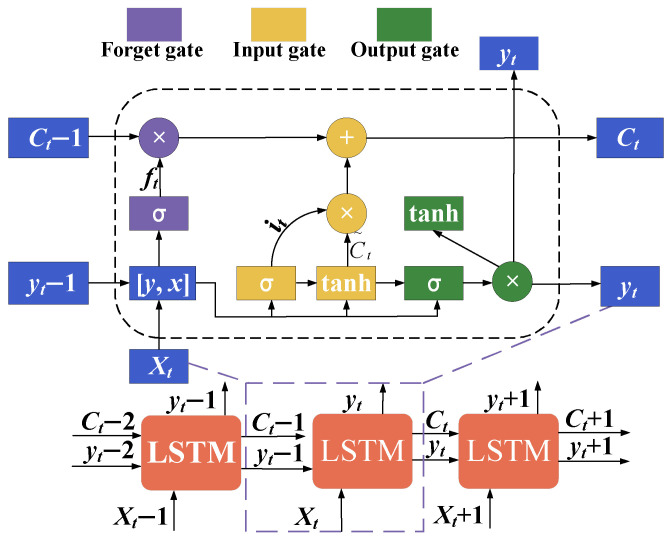
Schematic diagram of LSTM model cell structure.

Long Short-Term Memory (LSTM) is a special type of Recurrent Neural Network (RNN), and its basic structure is illustrated in Figure 3. At each time step, LSTM takes inputs, the hidden state, and the memory cell from the previous time step as input. The prediction process of LSTM can be summarized as follows [28]:

(7) $$f_{t}=\sigma (W_{f}\times y_{t-1}+R_{f}\times x_{t}+b_{f})$$

(8) $$i_{t}=\sigma (W_{i}\times y_{t-1}+R_{f}\times x_{t}+b_{i})$$

(9) $${\tilde{C}}_{t}=\tanh(W_{c}\times y_{t-1}+R_{c}\times x_{t}+b_{c})$$

(10) $$C_{t}=f_{t}\times C_{t-1}+i_{t}\times {\tilde{C}}_{t}$$

(11) $$o_{t}=\sigma (W_{0}\times y_{t-1}+R_{0}\times x_{t}+b_{0})$$

(12) $$y_{t}=o_{t}\times \tanh(C_{t})$$

where σ represents the activation function, and the expression $\sigma (x)={\left(1+e^{-x}\right)}^{-1}$; tanh represents the activation function of the hyperbolic tangent function, whose expression is $\tanh=\frac{e^{x}-e^{-x}}{e^{x}+e^{-x}}$. W, R, and b are the input weights, recurrent weights, and bias vector, respectively. $y_{t-1}$ represents the cell output at time step t−1, and $x_{t}$ represents the cell input at time step *t*.

### 2.6. 1DCNN-LSTM Prediction Model

**Figure 4:**
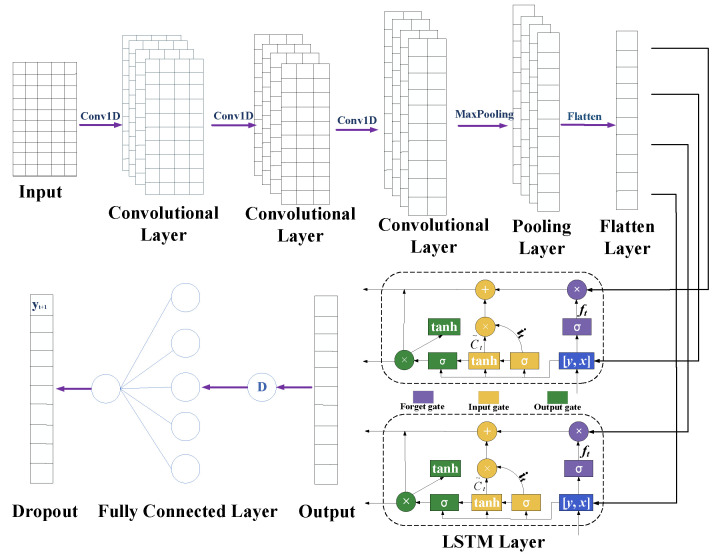
Schematic diagram of 1DCNN-LSTM structure.

In this section, the 1DCNN is integrated with the LSTM to develop a 1DCNN-LSTM model, with the objective of enhancing the precision in forecasting the pressure pulsation of centrifugal pumps. The 1DCNN functions as the feature extraction tool in this model, where it processes the data through its convolutional and pooling layers. This step is crucial for extracting relevant features and reducing the dimensionality of the data. Following this, the refined data from the 1DCNN is channeled as input into the LSTM. Within the LSTM network, the forget gate, input gate, and output gate dynamically refine their parameters through rigorous iterative training on extensive datasets. This continuous adjustment allows the LSTM to effectively manage and interpret the temporal characteristics of the data. The temporal relationships between the data are learned by the LSTM network through the information extracted by the 1DCNN network. This allows the LSTM to dynamically model the input–output data of the time series for accurate prediction. Finally, the fitted and trained data are output as predictions through a fully connected neural network in the 1DCNN-LSTM architecture [29]. The overall structure of the 1DCNN-LSTM prediction model is illustrated in Figure 4.

**Figure 5:**
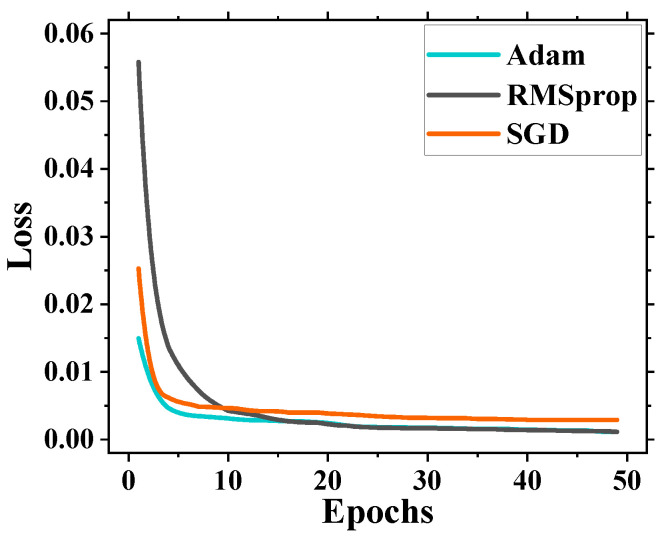
Training loss curve.

In order to select the most suitable gradient descent optimization algorithm for the 1DCNN-LSTM based on its structural characteristics, a comparative experiment was conducted using common optimization algorithms such as Adaptive Moment Estimation (Adam), Root Mean Square Propagation (RMSprop), and Stochastic Gradient Descent (SGD) [30]. The changes in their loss functions were analyzed to determine the optimal choice. The training loss curve, as shown in Figure 5, represents the Root Mean Square Error (RMSE) on the vertical axis and the number of epochs or iterations during training on the horizontal axis. From Figure 5, it can be observed that the Adam optimization algorithm reaches a stable loss function value around the 10th training iteration, while the stability of the RMSprop and SGD optimization algorithms lags behind. This indicates that the Adam optimization algorithm exhibits faster convergence speed when dealing with this time series data. Furthermore, by comparing the loss function values of the three optimization algorithms, it can be observed that the RMSE is the smallest for the Adam method. Based on the aforementioned analysis, the Adam optimization algorithm was selected because it demonstrated the best performance in training the model.

## 3. VMD-PSO-1DCNN-LSTM Centrifugal Pump Pressure

### 3.1. Model Workflow

**Figure 6:**
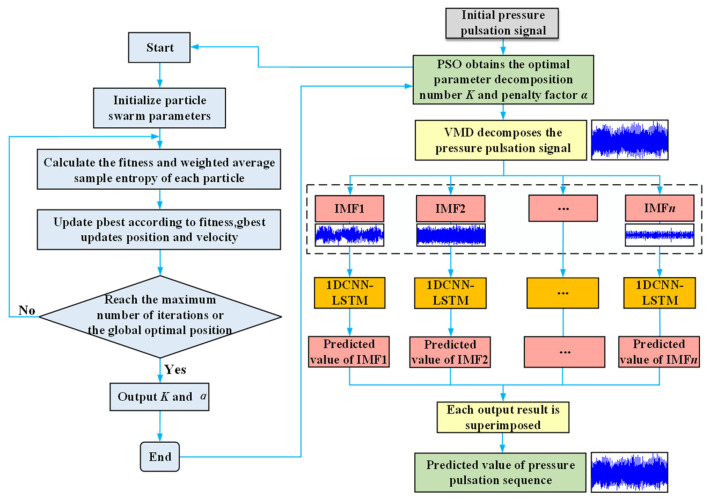
Workflow of the VMD-PSO-1DCNN-LSTM centrifugal pump pressure pulsation prediction model.

The workflow of the VMD-PSO-1DCNN-LSTM model is depicted in Figure 6, and the implementation steps are outlined as follows:

Step 1: The PSO Particle Swarm Optimization algorithm is employed to optimize the selection of VMD decomposition parameters by analyzing the convergence speed and stability of the convergence curve.

Step 2: The original pressure pulsation signal is decomposed using VMD with the selected parameters, resulting in multiple Intrinsic Mode Function (IMF) components.

Step 3: The obtained IMF components, which are time series data, are fed into the 1DCNN for feature extraction and dimensionality reduction through the processing of convolutional and pooling layers.

Step 4: The data processed by the 1DCNN is then utilized as input for the LSTM. The parameters of the forget gate, input gate, and output gate of the LSTM network are continuously adjusted through iterative training on a large amount of data. This enables the LSTM to learn the temporal relationships between the data from the information extracted by the 1DCNN network, thereby effectively dynamically modeling the input–output data of the time series for prediction and data analysis.

Step 5: The fitted and trained data are output as predictions through a fully connected neural network in the 1DCNN-LSTM architecture.

Step 6: The predicted results are compared with the actual data, and error metrics are calculated to evaluate the performance of the prediction model.

### 3.2. Evaluation Metrics

In the testing set, the original data was compared with the predicted results data, and the Root Mean Square Error (*RMSE*) and Mean Absolute Percentage Error (*MAPE*) were utilized as evaluation metrics.

(13) $$RMSE=\sqrt{\frac{1}{n}\times \sum_{i=1}^{n}{\left(y_{i}-{\hat{y}}_{i}\right)}^{2}}$$

(14) $$MAPE=\frac{1}{n}\times \sum_{i=1}^{n}\left|\frac{y_{i}-{\hat{y}}_{i}}{y_{i}}\right|\times 100\%$$

In the equations, n represents the number of samples, $y_{i}$ represents the true values, ${\hat{y}}_{i}$ and represents the predicted values.

## 4. VMD-PSO-1DCNN-LSTM Centrifugal Pump Pressure Pulsation Prediction

### 4.1. Centrifugal Pump Pressure Pulsation Experiment

The pump selected for the study was a single-stage, single-suction centrifugal pump, and its main technical parameters are listed in Table 1.

**Figure 7:**
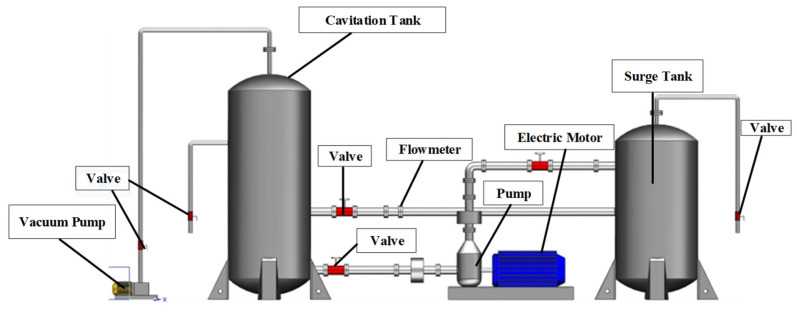
Schematic diagram of the closed-loop test rig.

**Figure 8:**
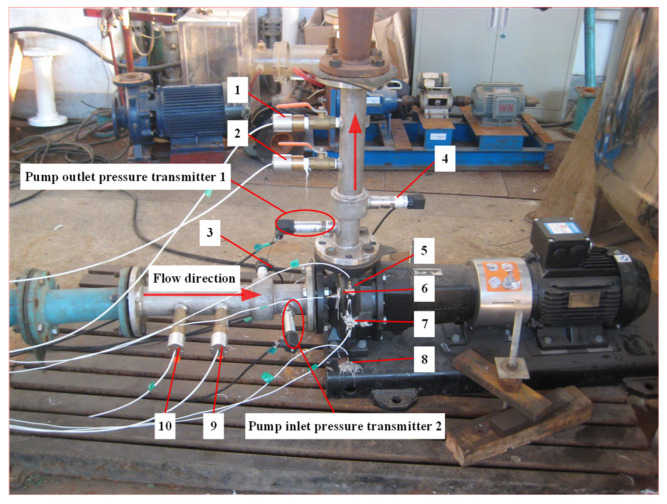
Experimental setup diagram. 1. Pump outlet hydrophone_1; 2. Outlet hydrophone_2; 3. Pump inlet pressure sensor_1; 4. Pump outlet pressure sensor_2; 5–7. X, Y, Z three-axis accelerometers; 8. Accelerometer; 9. Pump inlet hydrophone_2; 10. Pump inlet hydrophone_1.

A single-stage, single-suction centrifugal pump was selected in this study, which had the following main technical parameters: design head *H*_d_ = 20.2 m, design flow rate *Q*_d_ = 50.6 m^3^/h, design speed *n* = 2910 r/min, number of impeller blades *Z* = 6, and specific speed at the design operating point *n*_s_ = 132.2. The experimental part of the study was conducted on a closed-loop test rig, as depicted in Figure 7. Due to the significant differences in the pressure fluctuations at the inlet and outlet of the centrifugal pump, two pressure sensors with different ranges were used in this experiment. The position of the sensor is shown as “Pump inlet pressure transmitter 2” in Figure 8.

**Figure 9:**
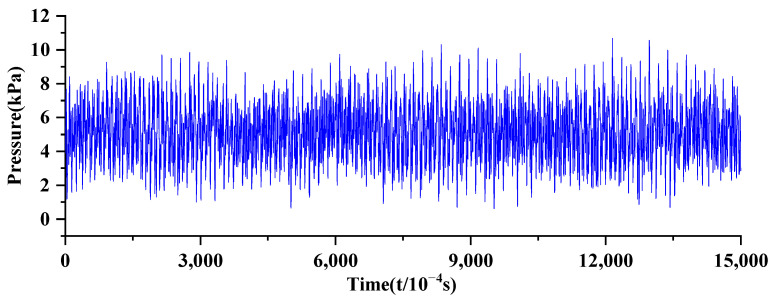
Original pressure fluctuation signal.

For the acquisition of pressure pulsation values, a high-frequency dynamic pressure sensor and a static pressure sensor, model HM90-H2-2-VCR-F-W2, were selected. The main performance parameters are shown in Table 2 and Table 3. The data from the sensor located at the inlet was selected for this study. The inlet pressure condition was set to 8 kPa, and the sensor sampling frequency was 10,000 per second. The current signals output by the pressure sensors were collected using a USB 6343 data acquisition card produced by NI Company (Austin, TX, USA). The original pressure fluctuation data used are illustrated in Figure 9.

### 4.2. VMD-PSO Parameter Settings

**Figure 10:**
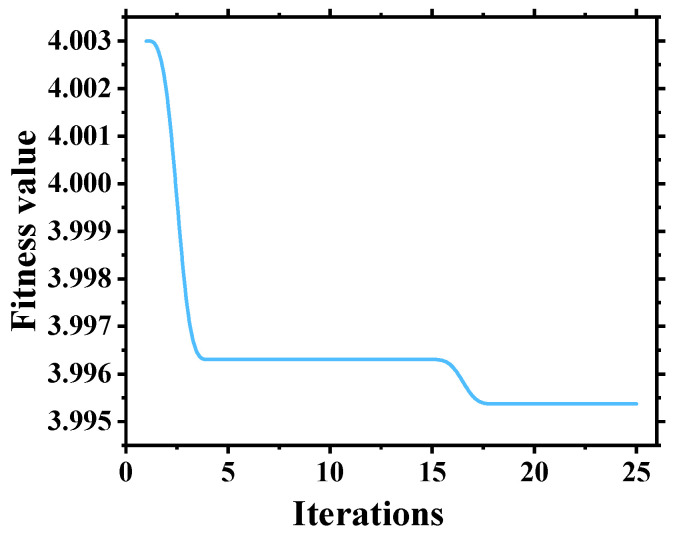
Convergence curve of VMD-PSO parameter optimization.

In the PSO algorithm, the values of $c_{1}$ and $c_{2}$ were set to 1.5, and w was set to 0.8. The initial velocities of particles were randomly generated within the range of [0, 1]. The population size was set to 10. After conducting the PSO optimization calculations, the optimal number of VMD decompositions was determined to be 6, the optimal penalty factor was found to be 532, and the optimal comprehensive index was measured as 3.9954. The convergence process is illustrated in Figure 8. The noise tolerance was set to 0, and the convergence criterion tolerance was set to 1 × 10^−7^. The convergence curve of VMD-PSO parameter optimization is shown in Figure 10.

### 4.3. VMD-PSO Parameter Settings

The configuration details for the 1DCNN-LSTM model utilized in this research are detailed in Table 4. Prior to model training, the data were subjected to several preprocessing steps, notably normalization, to ensure optimal model performance. Furthermore, the dataset was partitioned into training and testing subsets following the chronological sequence of the data, allocating 80% (12,000 samples) for training and the remaining 20% (3000 samples) for testing. To mitigate the risk of overfitting and enhance the model’s ability to generalize, a dropout rate of 0.2 was implemented.

### 4.4. VMD-PSO Preprocessing Comparative Analysis

**Figure 11:**
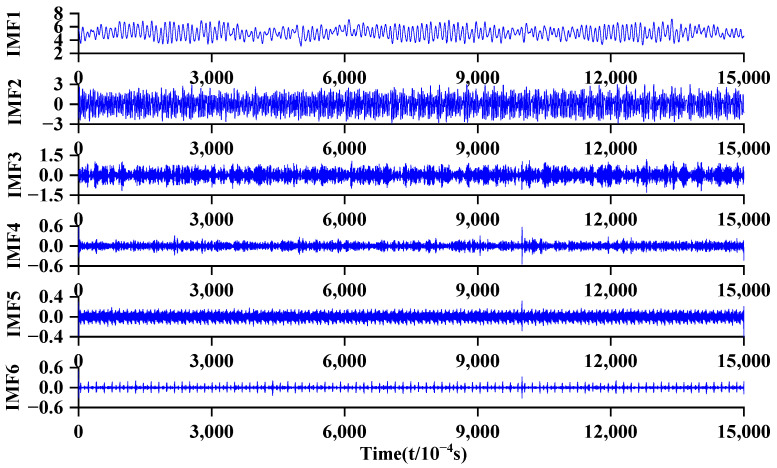
VMD signal decomposition graph.

Based on the theories mentioned in Section 2.1 and Section 2.2, the data points of the inlet pressure pulsation time-domain data of the centrifugal pump were subjected to PSO-VMD decomposition. The decomposition results of the original pressure fluctuation signal are shown in Figure 11.

**Figure 12:**
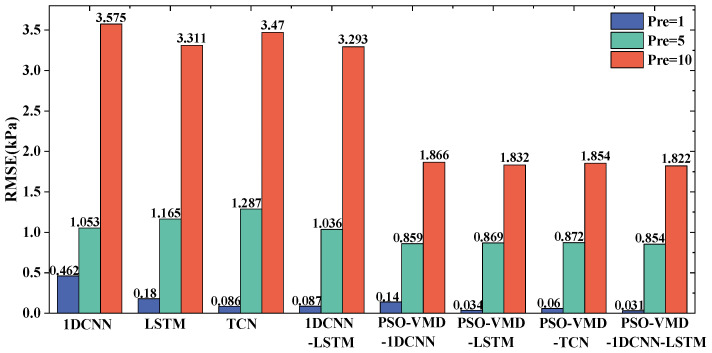
*RMSE* results of different prediction models.

**Figure 13:**
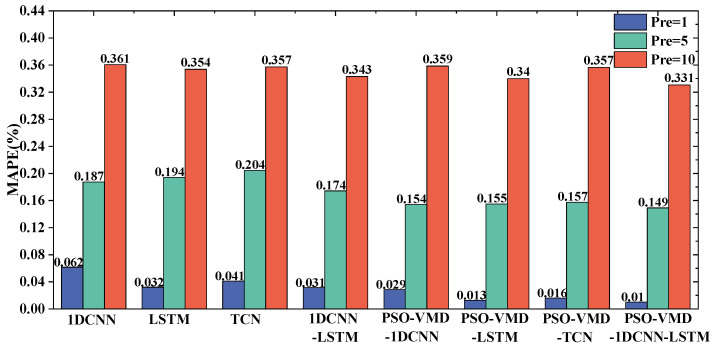
*MAPE* results of different prediction models.

To verify whether the data processed by VMD-PSO preprocessing is more conducive to data prediction by neural networks, the preprocessed data was input into four sets of models: 1DCNN, LSTM, TCN, and 1DCNN-LSTM. A comparison and analysis were conducted with four sets of neural networks that did not undergo any data preprocessing. The prediction errors of each model are shown in Figure 12 and Figure 13.

It can be observed that the neural network prediction results using the data preprocessed by PSO-VMD exhibit improvements in both *RMSE* and *MAPE* compared with the group without preprocessing, as shown in Figure 12 and Figure 13. The enhancement is particularly significant in terms of *RMSE*, indicating the effectiveness and adaptability of PSO-VMD in decomposing pressure pulsation signals and accurately extracting their modal components. PSO-VMD employs Variational Mode Decomposition to extract local modes of the pressure pulsation signal and optimizes the parameters through Particle Swarm Optimization. This approach enables the neural networks to learn higher-level feature representations. By using these modal components as inputs, the neural networks can better capture the global characteristics of the pressure pulsation signal. It is demonstrated that the combination of PSO-VMD and the four neural network models outperforms individual prediction models in terms of performance.

### 4.5. Comparative Analysis of Multiple Forward Prediction Steps

Based on the results of Section 4.4, it has been demonstrated that the combination of PSO-VMD and the four neural network models outperforms individual prediction models. To further validate the effectiveness of the proposed models in the prediction of centrifugal pump pressure pulsation, four models, namely PSO-VMD-1DCNN, PSO-VMD-LSTM, PSO-VMD-TCN, and PSO-VMD-1DCNN-LSTM, were designed and compared using multiple forward prediction steps. The forward prediction step size is defined as the time interval between the prediction target and the current time point used to make that prediction. Typically, after *n* prediction steps, the interval between the future value being predicted and the last data point used for the prediction is *n* time units. This means that at every point n steps ahead, the predicted value should closely match or conform to the actual observed value.

**Figure 14:**
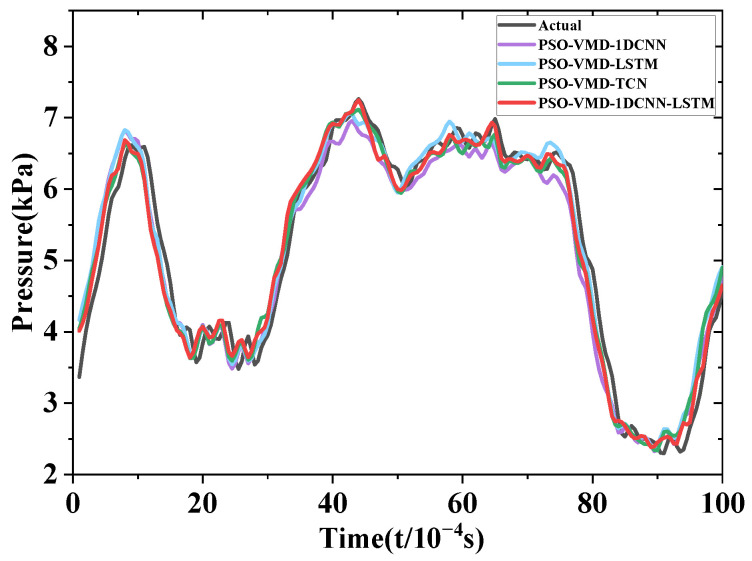
Comparison of prediction results for a forward prediction step of 1.

From the analysis of Figure 14, it can be observed that the prediction results of the four models closely align with the actual values when the forward prediction step is 1. By examining the *RMSE* and *MAPE* values presented in Figure 12 and Figure 13, it can be deduced that 1DCNN-LSTM exhibits reductions in *RMSE* of 0.10863 kPa, 0.00298 kPa, and 0.02853 kPa, as well as reductions in *MAPE* of 1.862%, 0.262%, and 0.61% when compared with the 1DCNN, LSTM, and TCN models, respectively. These findings indicate varying degrees of improvement in accuracy achieved by 1DCNN-LSTM. When considering this information along with Figure 14, it can be concluded that 1DCNN performs less effectively than the other three models, suggesting lower generalization performance in predicting centrifugal pump pressure pulsation. Both TCN and LSTM demonstrate similar prediction accuracy, closely resembling the actual values of centrifugal pump pressure pulsation. Furthermore, 1DCNN-LSTM shows a closer fit to the actual pressure pulsation curve with higher local alignment.

**Figure 15:**
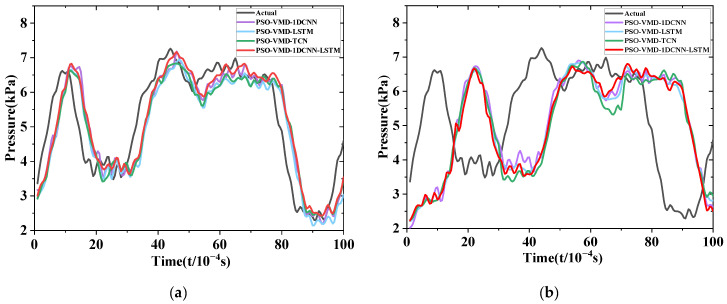
Comparison of prediction results for multiple forward prediction steps: (**a**) Pre = 5; (**b**) Pre = 10.

In the results with a forward prediction step of 5, reductions in *RMSE* of 0.00464 kPa, 0.01446 kPa, and 0.001787 kPa are observed for 1DCNN-LSTM compared with the 1DCNN, LSTM, and TCN models, respectively, as analyzed from Figure 12 and Figure 13. Additionally, 1DCNN-LSTM achieves reductions in *MAPE* of 0.502%, 0.591%, and 0.839% compared with the 1DCNN, LSTM, and TCN models, respectively. For the results with a forward prediction step of 10, 1DCNN-LSTM demonstrates reductions in *RMSE* of 0.043747 kPa, 0.009897 kPa, and 0.031267 kPa compared with the 1DCNN, LSTM, and TCN models, respectively. Moreover, 1DCNN-LSTM achieves reductions in *MAPE* of 2.775%, 0.922%, and 2.582% compared with the 1DCNN, LSTM, and TCN models, respectively. Through the analysis of Figure 15a, it can be observed that all four models exhibit similar trends to the actual values, but a local lag phenomenon is present. However, both 1DCNN-LSTM and TCN models show some differences compared with the 1DCNN and LSTM models in capturing local extrema, but they still effectively reflect the overall trend of the data. Through the analysis of Figure 15b, it can be deduced that all models exhibit significant differences from the true values, with all four models showing lagging and deviating phenomena. Specifically, the 1DCNN and 1DCNN-LSTM models capture individual peaks in local areas. Based on the performance of the models in forward prediction step sizes of 5 and 10, it can be concluded that 1DCNN-LSTM achieves higher prediction accuracy across multiple step sizes.

The error of the 1DCNN-LSTM model gradually increases with the step size due to the model’s limited ability to capture dependencies over larger prediction step sizes. As the forward prediction step size increases, potential errors from previous steps accumulate at each prediction step, and the uncertainty of predicting future values also increases with the expanded prediction range. Additionally, time series data contain complex nonlinear relationships and dynamic variability, which were difficult to fully capture by the model.

The 1DCNN-LSTM model demonstrates superior prediction results compared with the individual 1DCNN, LSTM, and TCN models. This is primarily due to the presence of complex nonlinear relationships in centrifugal pump pressure pulsation signals, which cannot be fully captured by linear models. The 1DCNN-LSTM model enhances its ability to model these nonlinear relationships by utilizing nonlinear activation functions and gating mechanisms. The convolutional operation in the 1DCNN layer and the gating mechanism in the LSTM layer effectively handle the nonlinear features in centrifugal pump pressure pulsation signals, resulting in improved prediction accuracy. In contrast, the linear operations in the 1DCNN, LSTM, or TCN models are less effective in capturing the nonlinear characteristics of centrifugal pump pressure pulsation signals. Another contributing factor to the improved performance of the 1DCNN-LSTM model is the presence of components with different frequencies and amplitudes in centrifugal pump pressure pulsation signals. The 1DCNN layer in the 1DCNN-LSTM model allows for multi-level feature extraction, enabling the capture of features at different time scales in centrifugal pump pressure pulsation signals.

This multi-level feature extraction effectively takes into account information at various scales, which cannot be fully considered by using a single 1DCNN, LSTM, or TCN model.

## 5. Conclusions

The PSO-VMD-1DCNN-LSTM model was proposed in this study to enhance the accuracy of pressure pulsation prediction. The centrifugal pump inlet pressure pulsation signals obtained from the experiment were decomposed, and the prediction model was analyzed and validated using experimental data. The following main conclusions are drawn:

(1) The PSO algorithm was utilized to optimize and select the experimental centrifugal pump pressure pulsation data, effectively mitigating the impact of nonlinearity and non-stationarity on prediction accuracy. The pressure pulsation data was further processed using Variational Mode Decomposition (VMD) with optimized parameters, enabling improved analysis and prediction.
(2) The combination of 1DCNN and LSTM neural networks, along with the Adam optimizer, enhanced the generalization capability and prediction accuracy of the neural network model.
(3) The experimental results demonstrated that when combined with the four neural network models, the proposed PSO-VMD outperforms the individual neural network prediction models in terms of prediction accuracy.
(4) Comparative tests revealed that the individual 1DCNN and LSTM models exhibit significantly lower prediction accuracy compared with the 1DCNN-LSTM model. This indicates that the incorporation of 1DCNN assists LSTM in capturing the temporal information of centrifugal pump pressure pulsation data. Moreover, the fusion model, 1DCNN-LSTM, effectively extracts essential features from industrial process data and adapts to the dynamic information within the data.
(5) The PSO-VMD-1DCNN-LSTM model consistently outperforms the PSO-VMD-1DCNN, PSO-VMD-LSTM, and PSO-VMD-TCN models in terms of prediction accuracy across various forward prediction steps, thus validating the accuracy and feasibility of the proposed model in predicting centrifugal pump pressure pulsation.

Our research has not yet achieved high accuracy in long-horizon forward predictions. In the next steps, we will conduct more in-depth studies. In future work, we will study the application of neural networks in hybrid models for transitioning from pressure fluctuation prediction to cavitation identification to enhance their applicability to practical engineering.

## Figures and Tables

**Table 1:** The main parameters of the NKG65-50-125 centrifugal pump are shown in [31].

Parameter	Symbol	Value	Parameter	Symbol	Value
Pump inlet diameter	$D_{s}$	65 (mm)	Blade outlet width	$b_{2}$	15.5 (mm)
Pump outlet diameter	$D_{d}$	50 (mm)	Volute base circle diameter	$D_{3}$	149 (mm)
Inlet diameter	$D_{j}$	79 (mm)	Volute inlet width	$b_{3}$	32 (mm)
Impeller outlet diameter	$D_{2}$	140 (mm)	Shaft frequency	$f_{0}$	48.5 (Hz)

**Table 2:** Main parameters of static pressure transmitters.

Measurement Location	Pressure Measurement Type	Measurement Range	Output Signal
Inlet pressure	Absolute pressure	0~1.6 bar	4~20 mA current
Outlet pressure	Relative pressure	0~4 bar	4~20 mA current

**Table 3:** Main parameters of pressure transmitters.

Measurement Location	Pressure Measurement Type	Uncertainty	Measurement Range	Output Signal
Inlet pressure	Relative pressure	±0.25%	±100 kPa	0~5 V (DC)
Outlet pressure	Relative pressure	±0.25%	0~1 MPa	0~5 V (DC)

**Table 4:** Parameter settings for 1DCNN-LSTM.

Method	Part	Item	Parameter Setting
1DCNN-LSTN	1DCNN	Layer1	1, 32, 7 × 7, 3
		Layer2	32, 64, 3 × 3, 1
		Layer3	64, 128, 3 × 3, 1
		Pooling method	Max-pooling
	LSTM	Input_size	512
		Num_layers	2
		Batch_first	true
		In_channels	1
		Hidden_size	200
		Out_size	1
	1DCNN-LSTM	Activation function	ReLU
		Optimization function	Adam
		Learning rate	0.001
		momentum	0.8
		Batchsize	64
		Epoch	30

## Data Availability

Data are contained within the article.
